# Robust Construction Safety System (RCSS) for Collision Accidents Prevention on Construction Sites

**DOI:** 10.3390/s19040932

**Published:** 2019-02-22

**Authors:** Byung-Wan Jo, Yun-Sung Lee, Rana Muhammad Asad Khan, Jung-Hoon Kim, Do-Keun Kim

**Affiliations:** 1 Department of Civil and Environmental Engineering, Hanyang University, Seoul 04763, Korea; joycon@hanmail.net (B.-W.J.); masadkhan87@gmail.com (R.M.A.K.); junghoon3301@hotmail.com (J.-H.K.); 2 Research and Development Center, YOUNGSHINE D&C, Gyeonggi-do 13487, Korea; Kimdokeun@daum.net

**Keywords:** collision accident, proximity warning system, heavy equipment, robust construction safety system

## Abstract

A proximity warning system to detect the presence of a worker/workers and to warn heavy equipment operators is highly needed to prevent collision accidents at construction sites. In this paper, we developed a robust construction safety system (RCSS), which can activate warning devices and automatically halt heavy equipment, simultaneously, to prevent possible collision accidents. The proximity detection of this proposed system mainly relies on ultra-wideband (UWB) sensing technologies, which enable instantaneous and simultaneous alarms on (a) a worker’s personal safety (personal protection unit (PPU)) device and (b) hazard area device (zone alert unit (ZAU)). This system also communicates with electronic control sensors (ECSs) installed on the heavy equipment to stop its maneuvering. Moreover, the RCSS has been interfaced with a global positioning system communication unit (GCU) to acquire real-time information of construction site resources and warning events. This enables effective management of construction site resources using an online user interface. The performance and effectiveness of the RCSS have been validated at laboratory scale as well as at real field (construction site and steel factory). Conclusively, the RCSS can significantly enhance construction site safety by pro-actively preventing collision of a worker/workers with heavy equipment.

## 1. Introduction

Occupational injuries occurring in the construction sector are a global issue, and the construction industry is a hazardous sector in which occupational accidents frequently occur [1]. In the last few decades, specifically in OECD (organization for economic cooperation and development) countries, despite various safety regulations executed by numerous industrial associations, the fatality rate of industrial accidents has been the highest compared to accidents in other similar industries [2]. In construction sites, the dynamic nature and simultaneous involvement of many resources in work zones leads to work zone complexity [3]. Such conditions can lead to collision accidents, incurring the risk of personal injuries. Among the several types of industrial accidents, collision accidents are the major cause of fatalities [4,5]. Recently, NOISH (National Institute for Occupational Safety and Health) has identified the common factors responsible for industrial collision accidents [6]. The most significant cause of collision accidents in work zones is the limited visibility or associated blind zone of heavy vehicles [7]. Therefore, there should be an active proximity detection and warning system to ensure the safety of workers from collisions.

In recent years, technological advancement and the latest tool development have paved a way for the development of active proximity warning systems (PWS). In this regard, wireless sensor networks have gained high popularity and have shown high potential for real applications in the construction field because of low-cost and high efficiency [8]. For instance, Zhu et al. [9] developed a proactive warning system to enhance the safety of workers at construction sites. This system can predict the next position of workers and mobile vehicles by observing their current and past locations. Park et al. [10] proposed a Bluetooth Low Energy (BLE)-based software programmable proximity system. This system has shown high accuracy under various approaching speeds of vehicles. An enormous amount of literature is presently focusing on the PWSs in heavy vehicles.

Generally, PWSs ensure collision safety using transmitters [RFID (Radio Frequency Identification), radar, or GPS (Global Positioning System), etc.] mounted on the heavy vehicles to detect in-range receivers (carried by workers) and an alert interface [11]. However, technological limitations hinder the use of PWS. For instance, application of vision technology such as cameras are limited to line-of-sight (LoS), and they can be easily blocked by various objects such as other equipment or material piles. In addition, camera-based proximity systems are unreliable under poor light conditions and images can be blurred by dust or fog. Moreover, alarms in radar systems can easily be triggered by various objects resulting in the generation of false alarms and extra fatigue to operators. 

For proximity warning at non-line-of-sight (NLoS), GPS and UWB (Ultra-Wideband) sensors have shown high applicability because of their high accuracy and suitability for outdoor applications [12]. UWB offers the advantages of high-speed data rate, high bandwidth, high accuracy, high-reliability, low-power, low-cost, short radio impulse, and high flexibility for the adoption of frequencies [13,14,15]. These benefits make UWB highly differentiable among other techniques and are the main causes of its high popularity in various industries. In fact, these technologies are expansive compared to other commercially available tools [16].

The main purpose of this study is implementing a robust construction safety system (RCSS) which activates an advanced warning device and automatic equipment control in order to prevent collision accidents from workers in hazardous positions. The RCSS utilizes a RFID sensor, around view monitor (AVM), and UWB sensor to recognize the workers and alert them to hazard situations using an alarm device. This system can automatically control the construction equipment when necessary. In addition, the RCSS was interfaced with a GPS technology and aimed at managing resources accessing construction sites. The rest of this paper is organized as follows. In Section 2, the literature on PWS is reviewed. In Section 3, the objective and scope for this paper is described. In Section 4, the operational scenario and implementation process of the RCSS is described. In Section 5, the performance evaluation methodology and results are presented. Finally, conclusions regarding this research are given in Section 6.

## 2. Research Trend

### 2.1. Proximity Warning System

Mine Safety and Health Administration (MSHA) studied more than 7500 surface mine accidents occurring between 1987 and 1996, and there were more than 120 fatalities caused by accidents. The most significant cause was the involvement of either moving or stationary power haulage machinery because of the associated blind spots of vehicles and non-availability of active warning systems [17]. The high number of fatalities clearly indicates the incapability of passive warning approaches such as signage, traffic control systems, and flaggers to ensure safety at construction and mining sites [18]. Therefore, it was proposed to update mining rules with mandatory warning systems on vehicles [17]. Since then, many researchers have focused on the development and implementation of active PWSs for both mining and construction vehicles. Ruff [19,20,21] at NIOSH evaluated various active technologies based on radar, sonar, infrared, GPS, and camera visions for proximity sensing and alert systems. These PWSs showed high applicability for construction as well as other working zones involved with the maneuvering of heavy vehicles.

Recently, many researchers have focused on the installation of RFID and magnetic field detection for providing real-time safety against collision accidents of vehicles at construction sites and mining areas [15,22,23,24,25,26]. HASARD [27] is an active proximity warning and alert system for heavy mining machines. It relies on magnetic sensing and works by measuring the strength of the magnetic field produced by the transmitter at the receiver. Teizer [28] introduced a reliable and an accurate magnetic field proximity system. This system warns both the operator and workers, simultaneously. Luo et al. [29] conducted a series of experiments at real construction sites and proposed an evaluation method for the responses of workers towards static hazard PWS. Recently, a hybrid (ultrasound and infrared) safety monitoring system [30] with a mobile sensing device has been proposed for the detection of workers at the dead zones of construction sites. The implementation of this system at construction sites resulted in significant reduction of accidents.

An advance system [25] with the utilization of three RFID tags has been introduced for the accident prevention of heavy machines and workers at construction sites. This system acquires data to estimate the size of the work area, collision risk at the construction site, and also generates warnings to individuals. Wu et al. [31] developed a low-cost, integrated, and autonomous ZigBee/RFID based system for real-time tracking of near-miss construction accidents. This system has shown high feasibility at construction sites through real applications. Another proactive system [32] based on ZigBee and RFID has been proposed to enhance safety at construction sites.

Similarly, GPS is a technology which has been extensively adopted to prevent equipment related accidents at construction sites and surface mines. GPS-based proximity and warning systems have shown high potential for reducing collision accidents in work zones [33]. Neito introduced a real-time software named VirtualMine [34] based on the integration of GPS technology, wireless network, and 3D graphics display to provide sufficient safety against collisions between workers and heavy vehicles at surface mines. In this system, the GPS tracks objects in real-time, the wireless network transmits data and issues proximity warnings, and the 3D display helps to create 3D maps in real-time. For construction safety and efficient project management at a dam site, a pro-active [35] system based on GPS has been introduced for collision avoidance. Moreover, a collision avoidance system [36] has been successfully tested at Teck’s Line Creek operation in British Columbia. This system utilizes GPS for proximity detection and warning and has shown capabilities to map outfitted objects such as overhead power lines, lighting plants, and stop signs.

### 2.2. Summary of Previous Research

For the prevention of collision accidents at construction sites, a pro-active proximity warning system to detect the presence of worker/workers, performing their duties in the close vicinity of heavy equipment, and to warn the operator is highly needed. Such proximity detection and warning systems can significantly enhance construction site safety by pro-actively preventing the collision of a worker with heavy equipment. Much research has been conducted to prevent hazardous collision accidents between workers and heavy equipment in construction sites [9,37,38,39,40]. Generally, proximity sensing technologies include ultrasound, radar, cameras, RFID, UWB, and GPS were used in the research. Researchers have found that various safety technologies can provide alerts to workers and heavy equipment operators when hazardous proximity situations exist. However, these proximity sensing technologies have specific limitations, such as weight, size, power source, reliability, applicability, and price. The main advantages and limitations of these proximity sensing technologies for prevention of collision accidents are shown in Table 1. Compared to other proximity sensing technologies, radio frequency (RF) technology (e.g., RFID and UWB) has unique advantages such as wide detection range, measurement rate, accuracy, obstacle classification, and immunity to influence from the surrounding environment (such as lighting, rain, or fog) [41]. In the implementation of proximity detection technology, it is very important to distinguish between nearby objects and workers, and it is also a major issue to accurately measure distance to workers approaching heavy equipment. In this regard, RF technology has a strong advantage for prevention of collision accidents compared to other proximity warning techniques.

## 3. Research Objective and Scope

**Figure 1:**
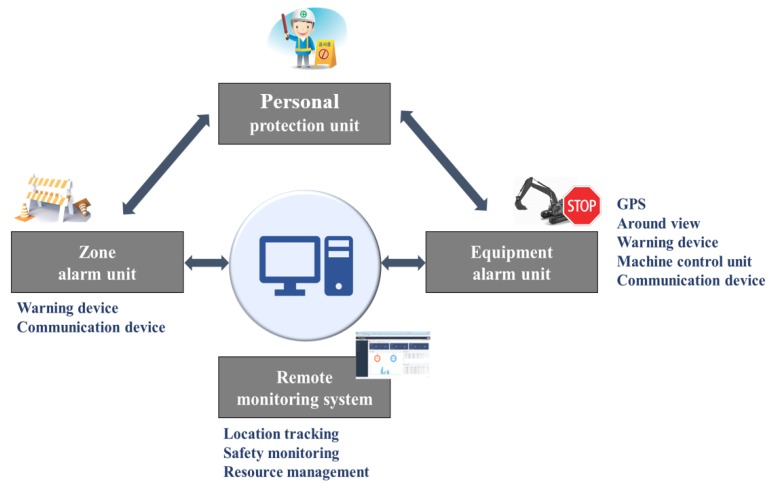
Concept of the robust construction safety system (RCSS).

**Figure 2:**
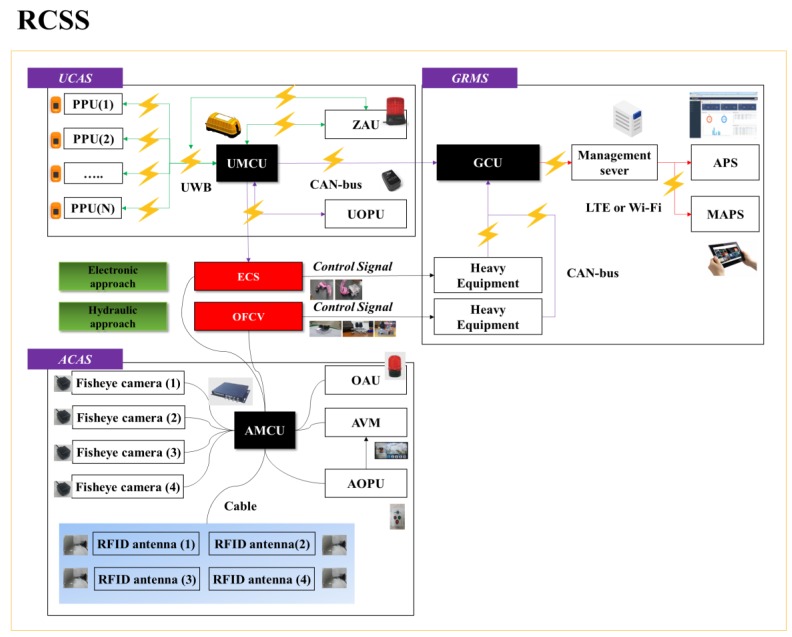
Overall hardware-software block diagram of RCSS.

In this study, we developed a RCSS that combines RFID, computer vision, UWB, and GPS technology. It monitors the movement of heavy equipment as well as workers’ movement at constantly dynamic construction sites to prevent potential accidents. The overall concept of the RCSS is shown in Figure 1. Figure 2 shows the overall hardware-software configuration of the RCSS. The complete framework of the proposed RCSS basically comprises of three separable sub-systems: i) AVM-based collision avoidance system (ACAS), ii) an UWB-based collision avoidance system (UCAS), and iii) a GPS-based resources management system (GRMS). The present study mainly focuses on the integration of the ACAS or UCAS with the GRMS to develop the RCSS. In the entire framework of the RCSS, each sub-system contributes its own share. The authors previously developed an ACAS as an initial step of this study. This ACAS was comprised of an AMCU (AVM main control unit), AOPU (AVM operator protection unit), RFID, and OFCV (oil follow control Valve). Further details of the ACAS can be found in [43]. The UCAS contains a UMCU (UWB main control unit), UOPU (UWB operator protection unit), PPU (personnel protection unit), ZAU (zone alarm unit), and ECS (electronic control sensor). The UMCU is installed on an excavator or forklift, whatever is the case. It utilizes UWB communication protocol to communicate with the PPU and ZAU. On the other hand, the UMCU utilizes CAN bus (controller area network) to communicate with the UOPU and GCU (GPS communication unit). While the GCU, APS (accident prevention solution), and MAPS (mobile accident prevention solution) are the separate interactive tools of the GRMS working under the RCSS.

The present study, for real demonstration, is limited to RCSS installation on excavators and forklifts, but it can be extended to all other construction equipment. Overall, communication in the RCSS is based on three types of communication protocols: i) UWB, ii) CAN-bus, and iii) GPS for onsite interaction of individual variables of a construction site (PPU, UMCU, UOPU, and ZAU). Details of communication decorum for the communication between various components are summarized in Table 2. There are several proximity warning technologies based on various communication protocols and approaches. Some features of common sensing technologies used in PWSs have been summarized in Table 3. It is not within the scope of this research to evaluate the relative effectiveness of the RCSS with existing proximity warning systems. The scope of the present study is limited to investigating the technical feasibility of integrating resource management and collision avoidance approaches using AVMs, proximity warning sensors, machine control, and GPS technologies to prevent collisions by heavy equipment at a construction site. Therefore, this study checks the feasibility and technical soundness of the RCSS for preventing collisions mainly focusing on excavators and forklifts.

## 4. Operational Scenarios of Proposed System

Major applications of the RCSS are as follows:

(a) Continuous and simultaneous monitoring of workers and construction equipment at construction sites;
(b) Real-time warning to workers, equipment operators, and entire working space as the level of risk increases;
(c) Automatic controlling of construction equipment before the occurrence of a collision accident.

The proposed system is robust in the sense it can be applied as a single unit or its sub-systems (GRMS, ACAS with GRMS, and UCAS with GRMS) can be applied separately, depending upon the requirements of construction sites. This section describes the operational scenarios and implementation process for each sub-system.

### 4.1. GRMS

**Figure 3:**
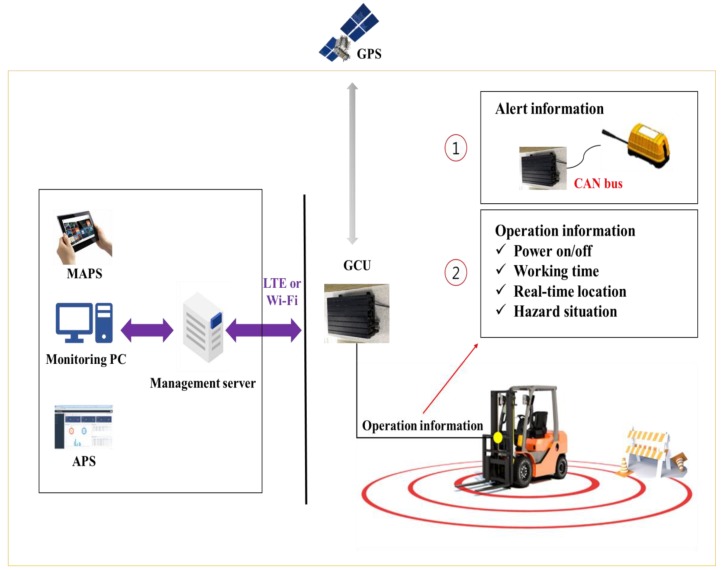
Data collection scenario.

In the GRMS, the lowest layer is implemented data collection and a communication device. A commercial communication device (GCU) has been adopted. In this device, GPS was chosen as the location tracking technology for construction equipment. The GCU uses two types of communication interfaces. The CAN-bus interface is for communication with the construction equipment or UMCU (in UCAS), and the LTE interface is for communication with the management server. Figure 3 depicts an operational scenario for data collection. Depending on the field environment, the data collection time can be set appropriately. The GCU connected to construction equipment collects operational information, as well as being able to collect alert information from the UMCU installed on the top of equipment. Then GCU transmits the collected information to the management server using LTE or Wi-Fi. Table 4 shows the specifications and collected data types of the commercial communication device.

**Figure 4:**
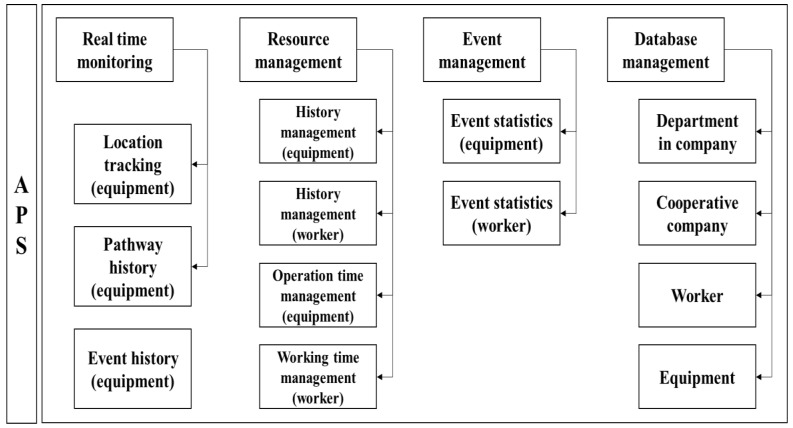
Accident prevention solution (APS) data architecture.

The APS is the main information management tool in the GRMS for general managers. The managers can access and manage information of construction resources via web browsers, including internet explorer (Microsoft) or chrome web browser (Google). The APS enables all the construction managers to log into a portal site and immediately obtain the necessary information for safety planning of a construction site. The APS enables input of field resources as list, real-time visualization of the tracked equipment in the monitoring window, signaling of upcoming hazards to the resources, statistics of event information, and history management of resources. Visualization technology of event data and location tracking of equipment enables managers to monitor the progress of the construction resources and have a more intuitive understanding of the safety status in the construction site. The APS data architecture is depicted in Figure 4.

### 4.2. UCAS and GRMS

The UCAS and GRMS consist of six devices: UOPU, PPU, ZAU, UMCU, ECS, and GCU. In this research, we selected commercial proximity warning sensors (UOPU, PPU, UMCU, and ZAU) as the real-time worker detection and alarm devices. These devices can be classified based on their functions and contribution in the UCAS.

Proximity warning devices: The proximity warning devices include UOPU, PPU, ZAU, and UMCU devices based on UWB and CAN bus interfaces. The UMCU can be mounted on the construction equipment using a flexible magnetic foot. The PPU can be mounted on the helmet of a construction worker and includes an alarm function. The appropriate warning distance can be set via the UOPU. The ZAU is installed on the construction site and provides both audible (speaker) and visual (LED) warning information when a detection signal is received from the PPU or UMCU. The ZAU is used to alert resources approaching hazard zones. The UOPU and UMCU communicate to each other using CAN bus. The UMCU interacts with the PPU or ZAU via the UWB communication. Whenever, either the PPU or ZAU receives a signal in a hazard zone, they automatically transmit the signals to the UMCU. The UMCU finally communicates with the UOPU. This real-time and sequential communication results in simultaneous triggering of an alert in all involved devices such as the PPU, ZAU, and UOPU. Thus, this system enhances the safety of the entire work zone by pro-actively generating an alert using proximity warning devices and effectively reduces collision accidents.

**Figure 5:**
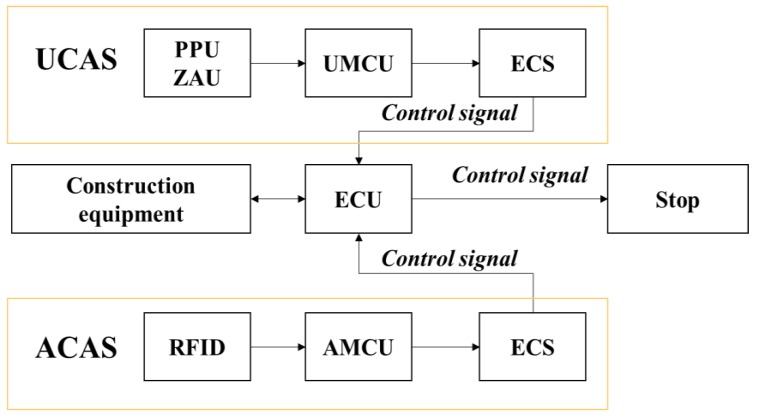
Mechanism of electronic control approach.

**Figure 6:**
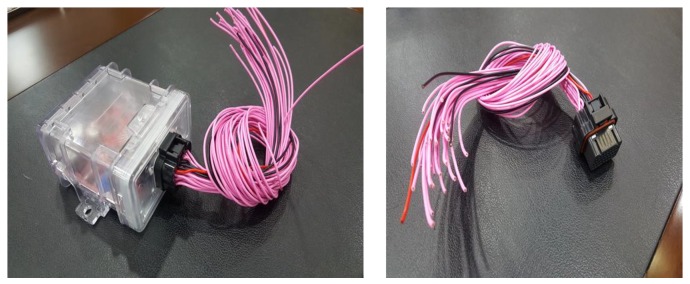
ECS.

ECS: In addition to UMCU communication with UPOU upon receival of a communication signal from the PPU or ZAU and also communicates with the ECS. The ECS activates the Electronic Control Unit (ECU) to control the equipment instantaneously. The basic techniques for hydraulic control of equipment was developed previously by the authors [43]. However, the hydraulic machine control demands the manufacturing and installation of an OFCV as well as modification of hydraulic line in the equipment. For this reason, it should be done only by trained engineers. Therefore, we applied a novel electronic control approach to overcome the technical disadvantages of hydraulic control. Recently, construction equipment such as excavators and forklifts is being converted from existing hydraulic control systems to electronic control systems. Construction equipment with electronic control systems can be controlled automatically using ECSs. The electronic control mechanism consists of four stages: i) detect workers in hazard zone (from UWB or RFID), ii) transmit recognition signal to ECS (from UMCU or AMCU), iii) transmit control signal from ECS to ECU of heavy equipment, and iv) control heavy equipment using ECU. All construction equipment with electronic control systems can be controlled in the same mechanism. Figure 5 depicts the electronic control mechanism, and Figure 6 shows the developed ECS.

**Figure 7:**
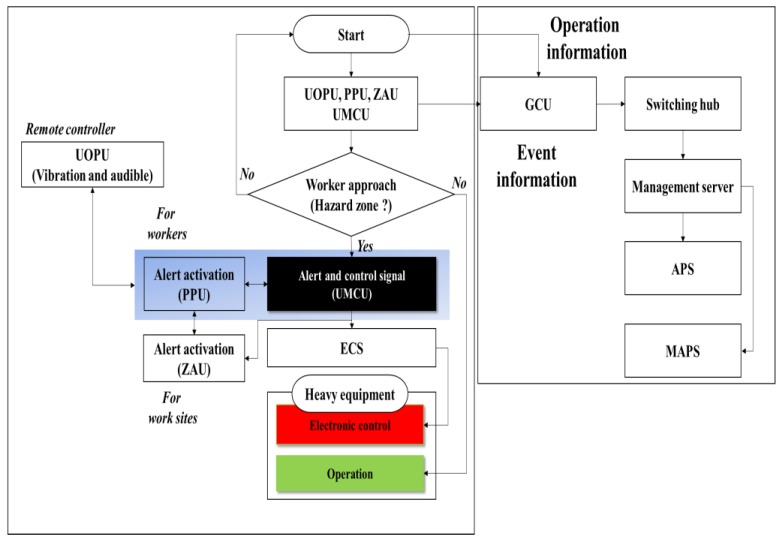
Operational scenario of UCAS and GPS-based resource management system (GRMS).

**Figure 8:**
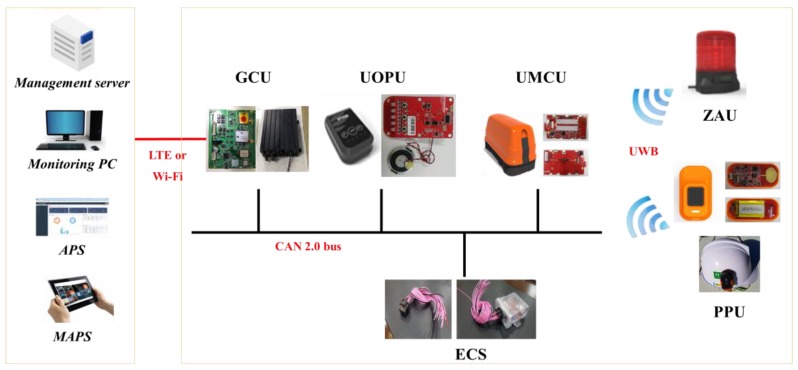
Hardware-software of UCAS and GRMS.

GCU: The UCAS is integrated with the resource management system through the GCU. The GCU is installed on all equipment present at a construction site, and it collects location and operation information of the heavy equipment, and alarm data, through a CAN-bus interface with the UMCU. The GCU transmits the collected data to the remote management server through the LTE or Wi-Fi interface. Figure 7 depicts the operation scenario of a UCAS and GRMS, and Figure 8 shows the overall hardware-software configuration. Table 5 lists the specifications of the PPU, UOPU, UMCU, ZAU, and ECS.

### 4.3. ACAS and GRMS

The ACAS consists of three main modules: i) an AVM module for visual monitoring, ii) RFID module for automatic detection of workers, and iii) AMCU module for equipment control. The ACAS was developed in a previous study [43], and the present study discusses the integration of the ACAS with the GRMS. An innovative OFCV was developed to control forklifts using a hydraulic control approach. Since a multiple PPUs (for all workers in a construction site) are required in the UCAS, the ACAS has an economical advantage over the UCAS (due to RFID tags being more cost effective than PPUs). Table 6 shows a previously developed OFCV for excavators and newly developed OFCV for hydraulic control of a forklift.

**AVM module:** This module consists of four fisheye cameras, one AVM, and an AOPU. The fisheye cameras were mounted around the construction equipment (one at the front, another at the rear, and two at each side of the equipment) to detect approaching workers. The AVM was mounted on the cabin of the construction equipment and allows the operator to see the surroundings view.

**RFID module:** The RFID module includes four RFID antennas and RFID tags. A RFID tag is deployed to each worker in the construction site. An RFID antenna was installed on the side or back of the excavator and emits radio frequency signals. The RFID antenna recognizes the RFID tags of workers approaching the excavator.

**AMCU module:** The operation of the equipment can be automatically controlled using an AMCU module. An OFCV is connected to the solenoid valve of the heavy equipment that controls the flow of oil for equipment operation. The heavy equipment can be automatically stopped using the OFCV. As mentioned in the UCAS, an excavator can be controlled using the ECS, which is backed in this operation by the ECU. In addition, each module including an OAU is combined in an AMCU.

**Figure 9:**
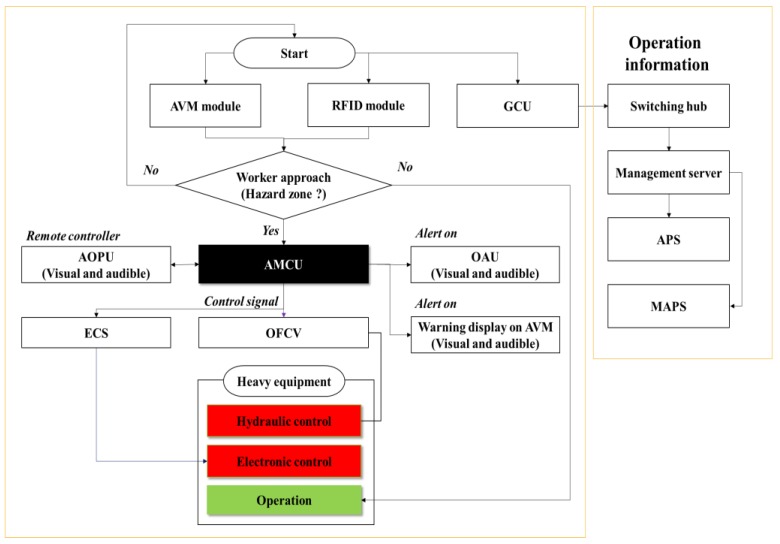
Operational scenario of ACAS and GRMS.

**Figure 10:**
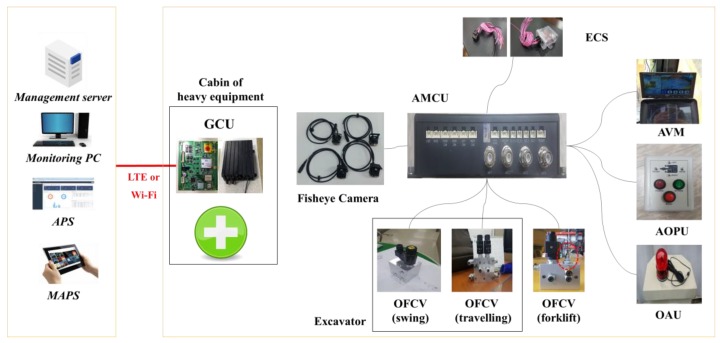
Hardware-software of ACAS and GRMS.

**GCU:** Like the UCAS, the ACAS is integrated with the GRMS through the GCU. However, only the operation and location information of heavy equipment is collected in this system. In order to collect additional information, it must be linked with an AMCU. This scope is not included in this study. All modules except for the GCU are connected to the AMCU by cables. Figure 9 depicts the operation scenario of an ACAS and GRMS, and Figure 10 shows the overall hardware-software configuration. Table 7 lists the specifications of the AVM, RFID, and AMCU.

## 5. Performance Evaluation

### 5.1. Laboratory Scale Tests

#### 5.1.1. Recognition Rate per 500 times

**Figure 11:**
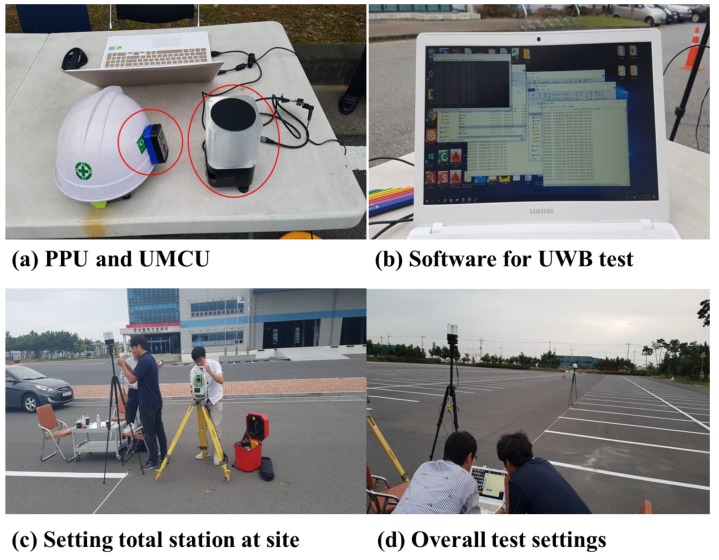
Test environment of recognition rate. (**a**) Pictorial view of PPU and UMCU, (**b**) software for UWB test, (**c**) setting total station at test site, and (**d**) overall setting of recognition test.

**Figure 12:**
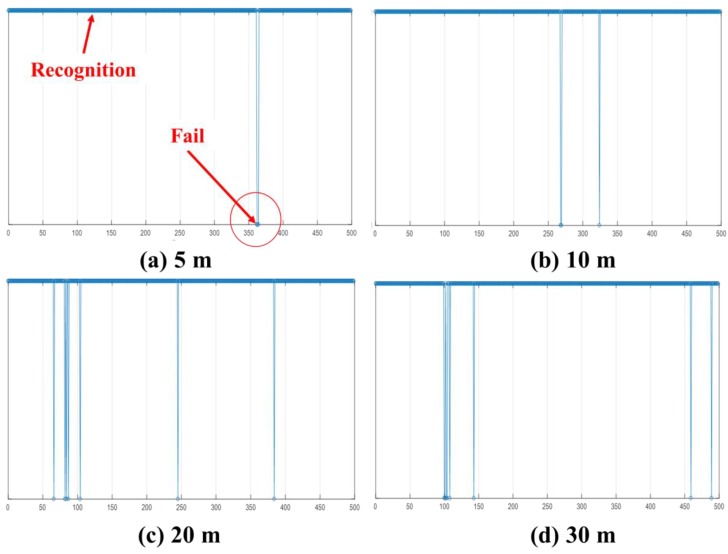
UWB communication between UMCU and PPU at (**a**) 5 m, (**b**) 10 m, (**c**) 20 m, and (**d**) 30 m.

The test of recognition rates for one-to-one UWB communication between the UMCU and another PPU in an outdoor environment were conducted by varying the distances between these two objects (Figure 11). The recognition rates in an outdoor environment were carried out at distances of 5, 10, 20, and 30 m apart. (Figure 12). Recognition rate is the number of times when the PPU was detected by the UMCU divided by the total readings (500 times) in this test. For accurate measurement of distances between the UMCU and PPU, a total station Leica TDRA6000 was used. 

For the cases when separation distances were 5 m and 10 m, the recognition rates were found to be 99.4%, which means that 497 times out of 500, the PPU was efficiently detected by the UMCU. The recognition rate slightly decreased to 98.6 % when the separation distance between the UMCU and PPU was increased to 20 m. When the separation distance between the PPU and UMCU was maintained at 30 m the recognition rate was found to be 98.4 %, which is still in an acceptable range (Table 8). From the interpretation of these results, it is easy to say that with the increase of distance between the UMCU and PPU, the recognition rate decreases. This is because of the fact that, generally, the strength of the signal becomes weaker as the PPU moves away from the UMCU. In this study, for effective detection, the proximity limit has been defined as 95% recognition rate or higher. These results helped in defining the warning zone around heavy machinery. The detection range of the proposed system can be modified according to the site-specific conditions. In this study, for experimentation, the proximity limit (warning zone) was set at 10 m as beyond this distance frequent alarms occur, causing extra fatigue to the operators. 

#### 5.1.2. Recognition Range Test

**Figure 13:**
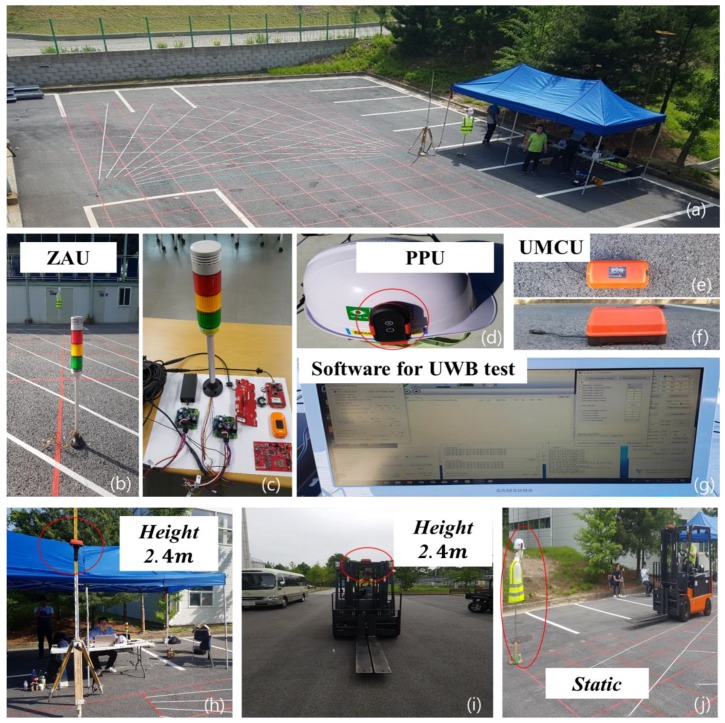
Experiment environment of recognition range.

In order to check the performance of the warning zone (10 m), we performed two separate experiments under different conditions: (a) stationary UMCU and moving PPU, and (b) stationary PPU and moving UMCU. The setup of these tests is shown in Figure 13. In Figure 13, (a) shows the overall area settings for the experimentation marked with radial lines and measured distances. These radial lines were separated at a constant interval of 10°. (b) represents a ZAU; in this experiment, ZAU has prime importance as it instantaneously activates the alarms whenever a PPU enters the detection range of a UMCU. (c) shows the prototype setting of the ZAU, PPU, and UMCU. (d) shows the safety helmet and holder with a PPU. (e) shows the top view of the UMCU. (f) shows the side view of the UMCU. (g) shows UWB recognition and distance check software. (h) shows the UMCU set on a tripod at a height of 2.4 m (stationary) from the ground so that it is at the same level as if installed on the top of a forklift. (i) shows a moving forklift installed with a UMCU at a height of 2.4 m. (j) shows a stationary mannequin wearing a safety helmet installed with a PPU at a height of 1.7 m (average height in Korea of a male worker). 

In this experiment, the real distance was obtained directly from the marked lines and compared with those obtained from the UWB software. The UMCU and PPU tests set the alarm position to 10 m and evaluated the accuracy of the position measurement. The UMCU was connected with a ZAU and its position was fixed. The static test of an approaching PPU to a stationary UMCU provides the detection area of the UMCU at various approaching rotation angles. The UMCU was installed using a tripod at the origin. The UWB signal was transmitted to record the distance at which recognition was successful, and distance against each detection point was recorded. 

(a) Stationary UMCU and moving PPU

**Figure 14:**
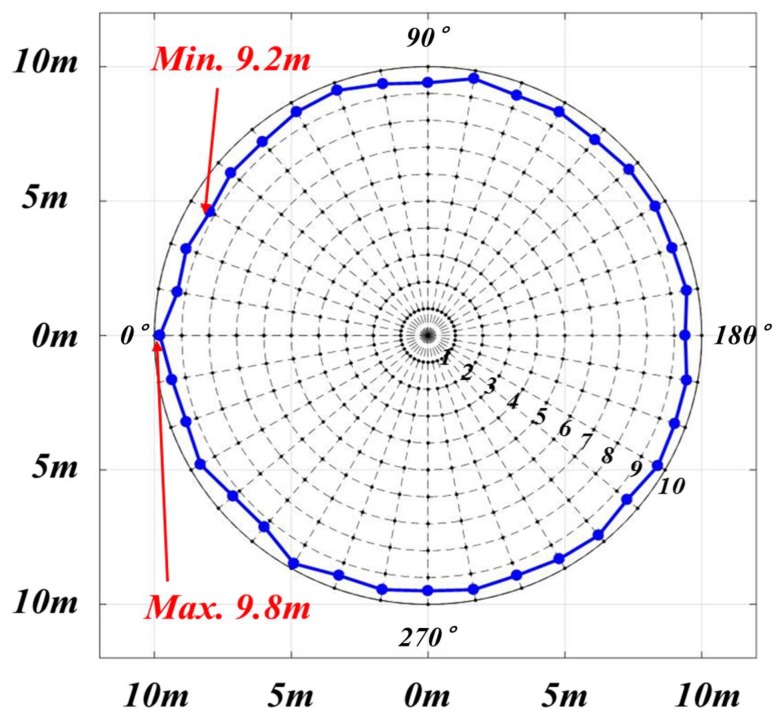
Result of static test.

In this test, the UMCU was held stationary at a height of 2.4 m (Figure 13(h)) and a mannequin wearing a helmet with a PPU attached was moved towards the UMCU along each radial line. The real distance of ZAU activation was checked against each angle. The results of this test have been summarized in Table 9. These results indicated the minimum distance of 9.2 m at rotation angle of 30°, whereas, the maximum detection distance was measured as 9.8 m at rotation angle of 0°(Figure 14). Against each radial line, 100 trials were performed to obtain the average distance. While compared with the UWB provided distance (10 m), the all-around error range of this system is from 0.2 to 0.8 m.

(b) Stationary PPU and moving UMCU

**Figure 15:**
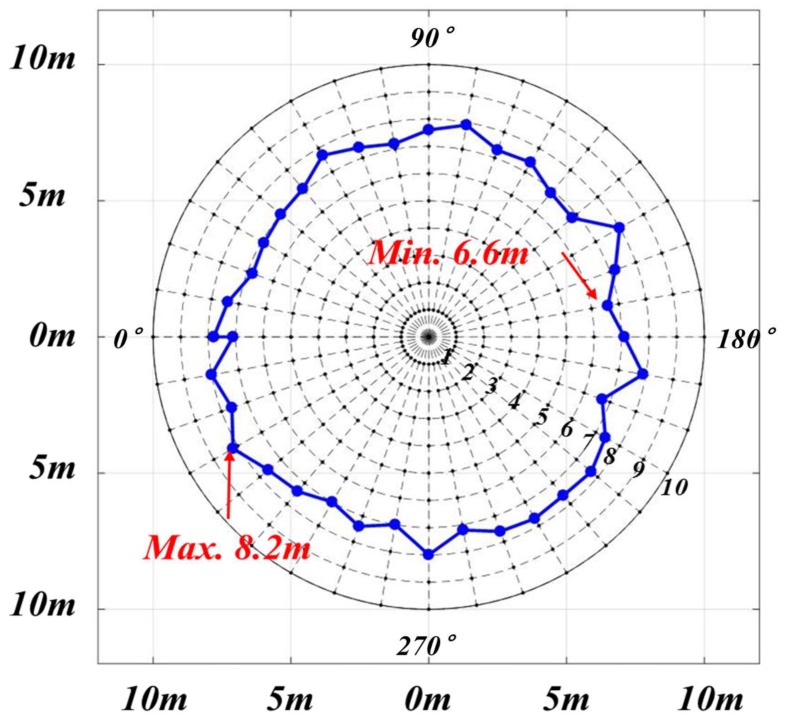
Result of dynamic test.

In this trial, an UMCU was installed on the forklift. Once again, the UMCU was fixed at a height of 2.4 m from the ground. A mannequin wearing a helmet with a PPU was placed 30 m from the forklift, and the forklift approached the mannequin at a constant speed (10 km/h). The results of this test have been summarized in Table 10. As the defined limit for activation of alarms was set at 10 m, in this test, instead of alarm activation at 10 m, it happened at less distance than defined. Minimum distance was found to be 6.6 m at a rotation angle of 170°, and the maximum distance of 8.2 m was measured at a rotation angle of 330°(Figure 15). However, in this case, the moving forklift affected for the reduction in the warning distance. According to operation speed requirements at construction sites, the managers can adjust the warning distance settings. From the test results, regarding the safety distance, the safety limits should be set at 10 m or 15m for the prevention of a collision accident.

### 5.2. Real Field Validations

The effectiveness on heavy machinery in actual field environments was checked at (a) construction site and (b) industrial factory (steel factory). In the following, each of the field experimentation sites has been explained separately.

(a) Construction site (Hwado-Yangpyeong expressway construction)

**Figure 16:**
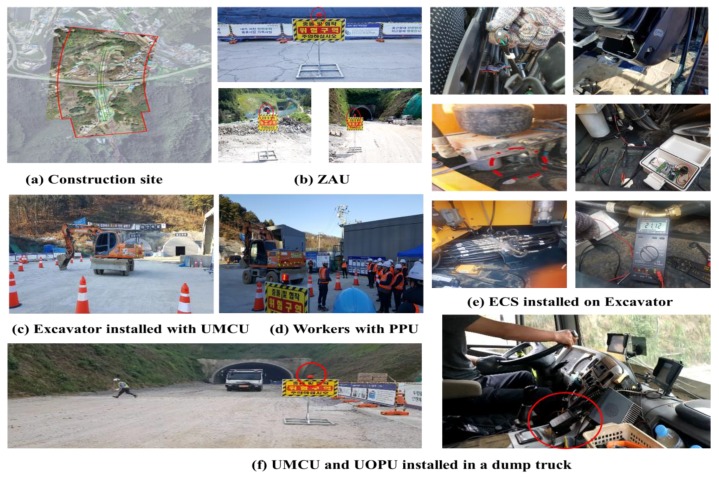
Field experiment environment (construction site).

After completing experimentation at laboratory scale, this study performed harsh field tests to further verify the applicability of the proposed total system mainly focusing on the UCAS and GRMS in an actual construction environment. For this purpose, the UMCU, PPU, UOPU, ZAU, ECS, and GCU were deployed at Hwado-Yangpyeong expressway construction site. At this construction site, a 4.92 km road was built including 3.92 km of a road tunnel. The challenging nature of this construction site provided an ideal dynamic environment to test the performance of the proposed system in construction site conditions. The long-term monitoring program started in July 2018 and continued until the end of December 2018. Figure 16(a) shows the plan view of the Hwado-Yangpyeong construction site. Figure 16(b) represents the arrangement of the ZAU installed at this construction site. Figure 16(c) illustrates an excavator installed with a UMCU, Figure 16(d) shows the workers with safety helmets installed with PPUs, Figure 16(e) demonstrates the ECS installed on an excavator, and Figure 16(f) depicts the UMCU and UOPU installed on a dump truck. The UOPU were activated correctly when a worker with a PPU approaching the UMCU and ZAU recognition range. Additionally, the alert function did not work when the worker was outside the recognition range of the UMCU. When the ECS was activated by the UMCU, the excavator (or forklift) was automatically stopped. The performance of the GRMS was evaluated for 6months (July 2018 to December 2018) at this construction site.

**Figure 17:**
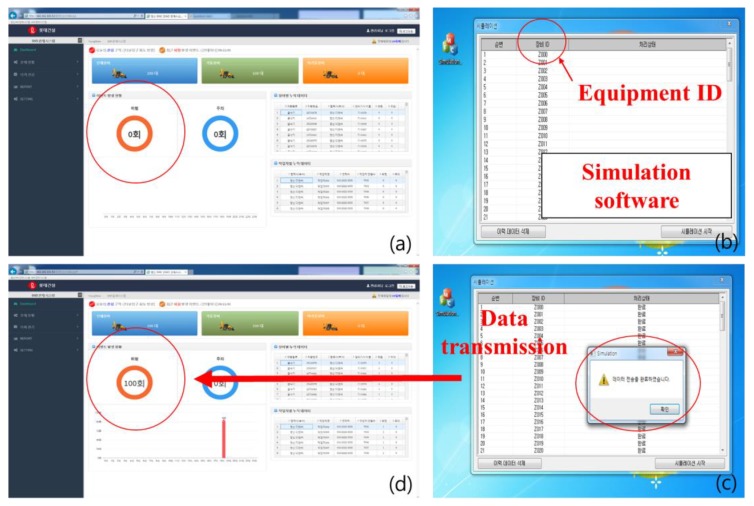
Test result of APS. (**a**) Initial settings, (**b**) equipment ID simulation, (**c**) equipment ID data transmission, (**d**) results in APS.

**Figure 18:**
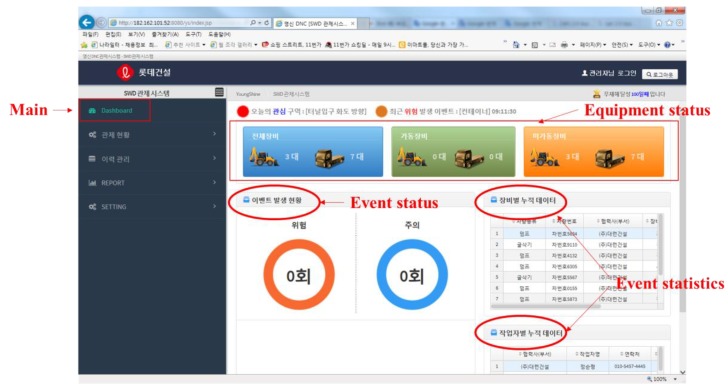
Main interface.

**Figure 19:**
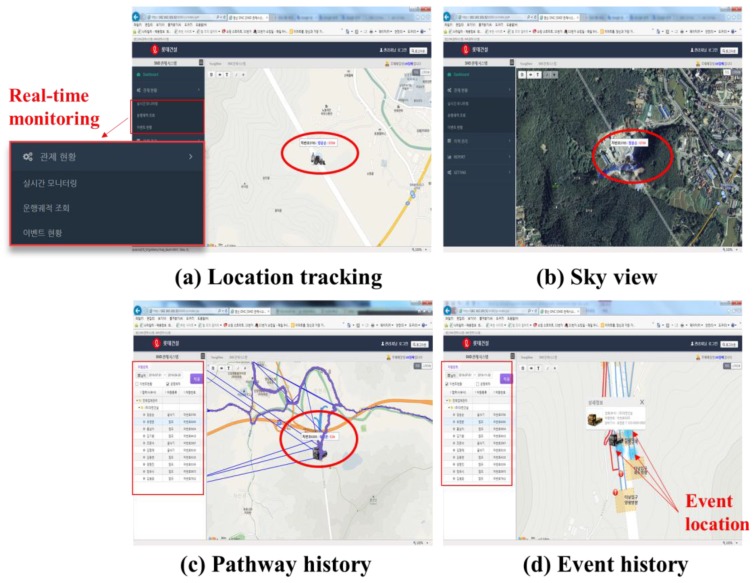
Real-time monitoring.

**Figure 20:**
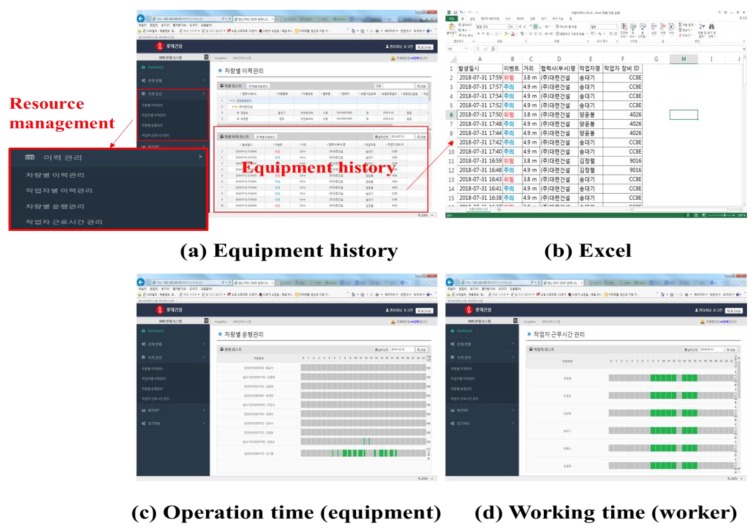
Resource management.

**Figure 21:**
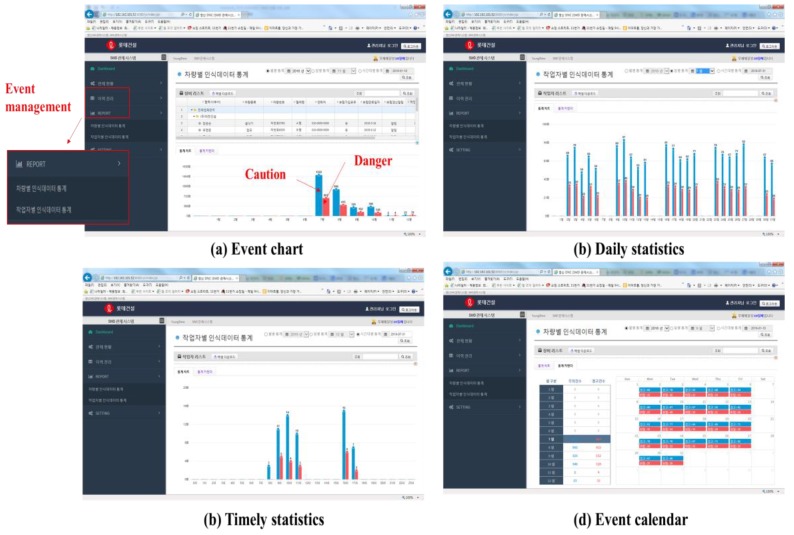
Event management.

Figure 17, Figure 18, Figure 19, Figure 20 and Figure 21 show the results of long-term construction site monitoring. The management server of the GRMS is a MySQL-based database server. It can simultaneously store and manage data from various construction equipment. As shown in Figure 17, the APS enables interaction of about 100 devices at the same time.

Figure 18 shows the main interface of the APS (first page). This dashboard is user friendly containing two operational windows. The left-hand window helps the users to select the appropriate options to get information about various resources. The window on the right hand shows Graphical User Interface (GUI)with the information of all the resources (total, operating, and stop). Moreover, it summarizes the equipment status, event status, and events history.

From the menu tool box, the second menu is for real-time monitoring, with shows GUI in the form of Daum maps. Figure 19a shows a 2D map of the working areas with information regarding resources, Figure 19(b) represents the sky view, (c) illustrates the pathway history, and (d) shows the event history. 

The third box in the menu box is related to resource management. This menu shows the operation time as well as the event status of equipment and workers using their device’s IDs, PPU and UMCU. The histories of the equipment and workers can be downloaded as Excel files (shown in Figure 20b). After selecting the third menu from the drop down options on the left-hand dashboard menu, the user can see the event management histories along with the daily statistics, time data, and event record on a calendar. The event management of the proposed system is shown in Figure 21.

**Figure 22:**
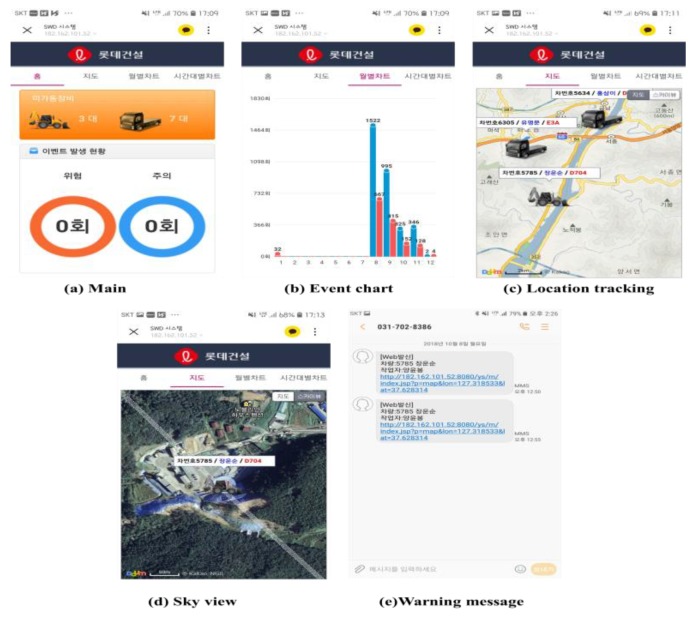
MAPS interface.

The interface of this system has also been provided as a mobile website. In this interface, it is possible to send event occurrence messages to the construction site managers, so that the manager can remotely check the safety status at construction sites using this MAPS. Screen shots of the MAPS are shown in Figure 22.

(b) Industrial factory application (steel factory)

**Figure 23:**
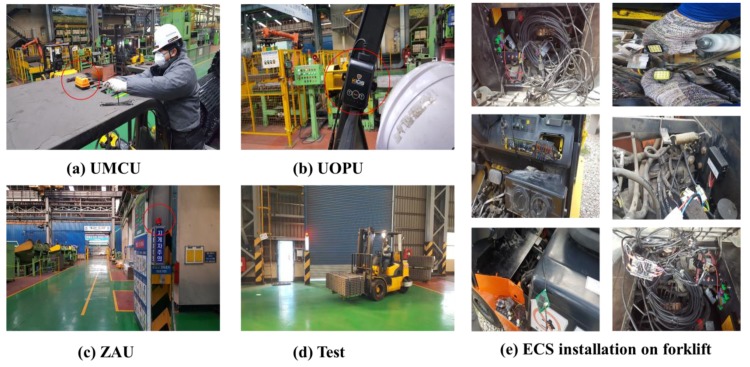
Field experiment environment (industrial factory).

Along with real field application at a construction site, we tested a sub-system of the proposed system at an industrial factory (steel factory) located in Pohang, South Korea. As mentioned above, our proposed system can be implemented as a total system or its sub-systems can be adopted separately. Usually, the use of forklifts is very limited at construction sites, therefore, we selected an industrial factory comprising mainly of forklifts as the main heavy machinery. In this field application, we implemented only a sub-system (UCAS) of our entire system. Figure 23a shows the installation of the UMCU at the top of a forklift used at steel factory, Figure 23b represents the UOPU attached inside the operators cabin of a forklift, Figure 23c illustrates the installation of ZAU at the side of the main walkway in the steel factory, Figure 23d shows a pictorial view of forklift motion during test, and Figure 23e depicts the various stages of installation of a ECS on a forklift.

## 6. Conclusions

Presently, the focus of the construction industry is to minimize accidents and occupational injuries at construction sites. The main purpose of this study was to determine if proximity sensing technology in the construction environment is feasible and to enhance the safety of the construction site by preventing collision with heavy equipment. For this goal, a robust construction safety system (RCSS) was developed by integrating resource management, a UWB-based proximity warning system, electronic machine control techniques, and GPS technologies to prevent collisions by heavy equipment at construction sites. The proposed system can be applied as a single unit or its sub-systems (AVM-based collision avoidance system (ACAS), an UWB-based collision avoidance system (UCAS), and a GPS-based resources management system (GRMS)), individually. In order to verify the system, this research investigated the feasibility and technical soundness of the proposed system mainly focusing on a UCAS and GRMS. The proposed real-time resource management, proximity warning, and machine control technology have proven to be effective in aiding the safety needs at a dynamic construction site. The UWB-based main control units can effectively detect the presence of workers approaching heavy equipment. Warning devices installed on excavators and dump trucks or forklifts successfully activated alerts in harsh field construction environments. 

Recent research of proximity warning systems have been performed by many researchers and this research has argued that the appropriate use of advanced proximity sensing technologies could be effective in preventing collision accidents. Conventional proximity warning technologies use RF technology to detect workers and activate alerts, monitor heavy machineries using GPS technology, manage light equipment using cameras, or operate individual systems for surrounding site resources. The proposed system can operate various technologies such as proximity warning using RFID, UWB, camera, and real-time position tracking using GPS technology as a single integrated construction safety system based on a GRMS. In addition, it is possible to apply sub-systems according to the conditions of the construction site. In addition, the RCSS can record safety data and important event data of equipment efficiently, and the collected data can be used for construction site safety planning.

Despite the various advantages listed above, the RCSS has the following limitations: workers and construction equipment must be installed and the specific conditions of the construction site can significantly affect the performance of the system. For example, RF technology can be affected by temperature, humidity, and ambient radio waves. Therefore, future research will analyze how these environmental factors affect the system, and it will include the efforts to improve the system.

## Figures and Tables

**Table 1:** Advantages and limitations of proximity sensing technologies [18,19,28,39,42].

Proximity Sensing Technology	Main Advantage	Limitation
Ultrasound	✓ Low initial cost ✓ Minimal device required ✓ Simplicity of installation	✓ Sensitive to the surrounding environment ✓ Limited detection range ✓ It requires direct line of sight ✓ Multiple sensors are required to cover the width of a large equipment
Radar	✓ Minimal device required	✓ It is difficult to distinguish between workers and other objects.
Camera (computer vision)	✓ Wide observation range ✓ It can easily distinguish a worker from other objects	✓ It’s performance depends on proper lighting ✓ Additional image processing technology required
RFID	✓ Capable of multiple antenna application ✓ Tag price is lower than UWB sensor	✓ Relatively limited detection range ✓ RFID tag should be provided to all objects for sensing
UWB	✓ Wide detection range ✓ Can function on outdoor and indoor ✓ It can easily distinguish a worker from other objects	✓ UWB device should be provided to all objects (worker or equipment)
GPS	✓ Can be used to location tracking	✓ Not functional indoors ✓ Not suitable for close range sensing

RFID: Radio Frequency Identification UWB: Ultra-Wideband GPS: Global Positioning System

**Table 2:** Communication protocols of RCSS.

Classification	Communication Protocol
PPU-ZAU	UWB
UMCU-PPU	UWB
UMCU-ZAU	UWB
UMCU-GCU	CAN bus
UMCU-UOPU	CAN bus
UMCU-ECS	CAN bus
GCU-server	LTE or Wi-Fi

PPU: Personal Protection Unit ZAU: Zone Alert Unit UWB: Ultra-Wideband UMCU: UWB Main Control Unit UOPU: UWB Operator Protection Unit CAN: Controller Area Network GCU: Global Positioning System Communication Unit LTE: Long-Term Evolution ECS: Electronic Control Sensor

**Table 3:** Comparison of the characteristics of proximity sensor technologies [19,42].

Characteristics	GPS	Radar	Magnetic Sensing	Ultrasound	RFID	UWB
Detection range	Long	Short/Medium	Short	Short	Short/Medium	Long
Accuracy of data	DE*	Medium	Low/Medium	Low/Medium	Medium	High
Update rate	High	High	High	High	High	High
Two-way alert	Yes	No	Yes	No	Yes	Yes
Installation and setup difficulty	Low/Medium	Low/Medium	Medium	Low/Medium	Low/medium	Low
Relative frequency of false alarms	Low	Medium	Low	Medium	Low	Medium
Tolerance to mud, dust etc.	High	Medium	High	High	High	Medium
Cost	Low/Medium	Low/Medium	Medium/High	Low	High	Low

DE*dependent on environment condition.

**Table 4:** Specifications and data types of GCU.

Classification		Factors
**GCU**	Interface 1	CAN 2.0 bus
	Interface 2	LTE(WCDMA)
	Location tracking	GPS
	Data type 1	Timestamp (for GMT and local time)
		Time offset (e.g.: GMT+00:00)
		GPS coordinates
		Status of GCU
		Status of equipment
	Data type 2 (with UCAS)	ID of UMCU / PPU / ZAU
		Event(eq. alert stats)
	Design	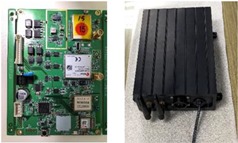

* GMT: coordinated universal time.

**Table 5:** Specifications of UMCU, PPU, UOPU, ZAU, ECS, and GCU.

Classification		Factors
UMCU	Size Interface 1 Interface 2 Mount Power UWB Frequency Bandwidth	177.8×84.1×86 (mm) CAN 2.0 bus UWB Flexible magnetic foot 12/24V 3993.6MHz 500MHz
PPU	Size Interface Mount Battery Alert type UWB frequency Bandwidth	40×72×19.5 (mm) UWB Helmet holder or belt clip 950 mAh Vibration and buzzer 3993.6MHz 500MHz
UOPU	Size Interface Alert type Power	93×130.5×47.5 CAN 2.0 bus LED and buzzer 12/24V
ZAU	Size Interface Interface Alert type Mount Power UWB frequency Bandwidth	122.7 x 140.6(H) x 111.5 (mm) UWB CAN 2.0 bus LED and buzzer Flexible magnetic foot 12/24V 3993.6MHz 500MHz
ECS	Interface Power	CAN 2.0 bus 12/24V

**Table 6:** Oil follow control valves (OFCVs) of three type specifications for hydraulic machine control.

Classification	OFCV Type		
	Excavator (Swing)	Excavator (Travelling)	Forklift
Max. Flow	32 lpm	35 lpm	35 lpm
Max. Pressure	350 bar	210 bar	210 bar
Operating hydraulic oil	ISO VG16 ~VG68	ISO VG16 ~VG68	ISO VG16 ~VG68
Design	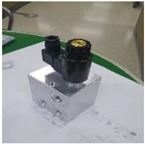	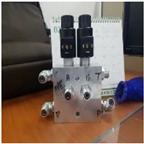	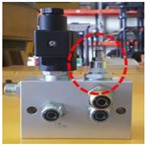

**Table 7:** Specifications of ACAS.

Classification		Factors
AVM	Display Resolution CPU processor UI Interface Alert type	HDMI, LVD8 MAX 1280×720 Dual Core 1.5 GHz Touch Screen CAN 2.0 bus Buzzer / Visual
Fisheye camera	Angle	V100°, H180°
AOPU	Alert type	LED and buzzer
OAU	Alert type	LED and buzzer
RFID antenna	Size Weight	130×100×20 (mm) 295 g
RFID tag	Type	ISO-1800-6C
AMCU(MC board)	Operation power Current Consumption	24 ~ 32 V 2A(MAX. 24V)
AMCU(AVM board)	Input	4 Port (Fisheye camera)
AMCU(RFID board)	Frequency Port	917.3–920.3 MHz 4 Port (RFID antenna)

**Table 8:** Test result of recognition rate.

Distance(m)	Total	Fail	Success	Recognition Rate (%)
5	500	3	497	99.4
10	500	3	497	99.4
20	500	7	493	98.6
30	500	8	492	98.4

**Table 9:** Test result of recognition rate.

Angle	Distance	Angle	Distance	Angle	Distance	Angle	Distance
0°	9.8m	100°	9.7m	190°	9.5m	280°	9.6m
10°	9.3m	110°	9.5m	200°	9.6m	290°	9.5m
20°	9.4m	120°	9.6m	210°	9.7m	300°	9.8m
30°	9.2m	130°	9.5m	220°	9.5m	310°	9.3m
40°	9.4m	140°	9.6m	230°	9.7m	320°	9.3m
50°	9.4m	150°	9.6m	240°	9.6m	330°	9.6m
60°	9.6m	160°	9.5m	250°	9.5m	340°	9.4m
70°	9.7m	170°	9.6m	260°	9.6m	350°	9.5m
80°	9.5m	180°	9.4m	270°	9.5m	360°	9.8m
90°	9.4m						

**Table 10:** Real distance of PPU detection by UMCU (dynamic test).

Angle	Distance	Angle	Distance	Angle	Distance	Angle	Distance
0°	7.8m	100°	7.9m	190°	7.9m	280°	7.0m
10°	7.4m	110°	7.3m	200°	6.7m	290°	7.4m
20°	6.8m	120°	7.4m	210°	7.4m	300°	7.0m
30°	6.9m	130°	6.9m	220°	7.7m	310°	7.4m
40°	7.0m	140°	6.8m	230°	7.6m	320°	7.6m
50°	7.1m	150°	8.0m	240°	7.7m	330°	8.2m
60°	7.7m	160°	7.2m	250°	7.6m	340°	7.6m
70°	7.4m	170°	6.6m	260°	7.2m	350°	8.0m
80°	7.2m	180°	7.1m	270°	8.0m	360°	7.8m
90°	7.6m						
